# Modular Architecture of Retinal Layers in Diabetic Patients Without Retinopathy

**DOI:** 10.7759/cureus.77657

**Published:** 2025-01-19

**Authors:** Pratyusha Ganne, Ganne Chaitanya, Suresh Vaikkakara, Arti Gupta, Rakesh U K

**Affiliations:** 1 Ophthalmology, All India Institute of Medical Sciences, Mangalagiri, Guntur, IND; 2 Neurology, Texas Institute of Restorative Neurotechnologies, University of Texas Health Science Center, Houston, USA; 3 Endocrinology and Diabetes, All India Institute of Medical Sciences, Mangalagiri, Guntur, IND; 4 Community and Family Medicine, All India Institute of Medical Sciences, Mangalagiri, Guntur, IND; 5 General Medicine, All India Institute of Medical Sciences, Mangalagiri, Guntur, IND

**Keywords:** diabetic retinopathy, modularity, oct (optical coherence tomography), pre-clinical, retinal layer thickness

## Abstract

Purpose

Diagnosing diabetic retinopathy (DR) in the pre-clinical stage is crucial to reversing DR. This study aimed to compare the retinal thickness changes between healthy controls (HCs) and diabetics without retinopathy (DWORs). For the first time, we would like to introduce the concept of network modularity analysis in studying retinal networks to demonstrate disrupted retinal layer organization as evidence of subclinical retinopathy.

Methods

This was a cross-sectional study on 156 eyes of HCs and 78 eyes of DWORs. Retinal layer thickness was measured on Spectralis OCT (Heidelberg Engineering, Heidelberg, Germany). Average thickness values from the outer ring of the ETDRS grid (Avg_O) and the inner ring (Avg_I) were calculated for each layer. Mean retinal thicknesses for each layer between the two groups were compared using the t-test. Age-related thickness changes were compared between the groups using Fisher's r-to-z transform. Group-based structural covariance networks were estimated for both DWORs and HCs. Optimal community architecture was estimated using Louvain’s modularity.

Results

Inner retinal layers, namely RNFL_C (HC: 10.16 ± 2.48 µm versus DWOR: 10.85 ± 2.23 µm; p=0.023) and INL_Avg_I (HC: 39.9 ± 3.7 µm versus DWOR: 40.9 ± 3.16 µm; p=0.035), were thicker in the DWOR group compared to the HC group. Outer retinal layers, namely OR_C (HC: 89.9 ± 3.8 µm versus DWOR: 88.7 ± 3.6 µm; p=0.017) and OR_Avg_I (HC: 81.4 ± 3.16 µm versus DWOR: 80.5 ± 2.28 µm; p=0.02), were thinner in the DWOR group compared to the HC group. The central sub-field showed an age-related thickening in retinal nerve fiber layer (RNFL) (r=0.117, p=0.04), GCL (r=0.078, p=0.17), inner plexiform layer (r=0.137, p=0.01), inner nuclear layer (INL) (r=0.29, p≤0.001), outer plexiform layer (r=0.256, p<0.001), and outer nuclear layer (r=0.197, p=0.001) layers in the HC group, which was not seen in the DWOR group. There was an abnormal increase in modularity among DWORs compared to HCs (Qhc=0.47, Qdowr=0.51, p=1.6x10^-8^). In the DWOR group, we noted a disruption in the community architecture and minimal inter-community interactions compared to HCs.

Conclusion

RNFL and INL are thicker in DWORs compared to HCs. Outer retinal layers are thinner in DWORs compared to HCs. On modularity analysis, we noted a disruption in the community architecture in the DWOR group compared to the HC group.

## Introduction

Diabetic retinopathy (DR) was long considered a micro-vasculopathy, but recent evidence has shown that neuroglial degeneration (reactive gliosis, reduced neuronal function, and neural-cell apoptosis) occurs before microangiopathy in animal models of DR and in the retina of diabetic donors [1,2]. Progressive ganglion cell and astrocyte loss is induced by diabetes that is related to inflammation, excitotoxicity, and oxidative stress [3]. As a consequence of this degeneration, we would expect a change in the thickness of the retinal layers. Previous studies to detect pre-clinical retinopathy using spectral domain optical coherence tomography have shown conflicting results with some studies showing thinning of inner retinal layers [4-6] in diabetics without retinopathy (DWOR)/mild non-proliferative diabetic retinopathy (NPDR) groups, while other studies have shown no difference in inner retinal thickness between the groups [7].

The retina is a complex neural network, with inter-layer interactions contributing to its functional integrity. Due to this interaction at a functional level, changes in the structure of one layer can affect the structure of the remaining layers. For example, in diseases such as glaucoma, degeneration of ganglion cells results in the thinning of not only the ganglion cell layer (GCL) but also the inner plexiform layer (IPL) and retinal nerve fiber layer (RNFL) [8].

In this study, we aim to compare the thickness of retinal layers between the DWOR and healthy control (HC) groups and study the age-related changes in the thickness of different layers between the groups. For the first time, we would like to introduce the concept of network modularity analysis in studying retinal networks to demonstrate disrupted retinal layer organization as evidence of subclinical retinopathy.

A pilot data related to the article was previously presented as a meeting abstract at 14th Asia-Pacific Vitreo-retina Society (APVRS) Congress in December 2021.

## Materials and methods

This was a cross-sectional observational study conducted between January 1, 2023, and November 1, 2023, at the All India Institute of Medical Sciences, Mangalagiri, Guntur, Andhra Pradesh, India. The study was conducted in accordance with the declaration of Helsinki and was approved by the Institutional Review Board (IEC Number: AIIMS/MG/IEC/2022-23/204). We included subjects aged > 18 years, with both type 1 and type 2 DM, a refractive error of not more than ±2 diopter sphere, and normal retinal examination. We excluded patients with any history or evidence of any systemic illnesses other than diabetes (neurological diseases, pregnancy, autoimmune diseases, etc.), which can affect retinal thickness values, those with any history of ocular surgery within the past six months, those with eyes with intraocular pressure (IOP) more than 21 mmHg, and those with signal strength of optical coherence tomography (OCT) scans <25 dB. A total of 156 eyes of HCs and 78 eyes of DWORs were included.

Sample size

According to a previous study [9], the mean inner retinal thickness was 91.6 μm in the DWOR group compared to 96.2 μm in the HC group. Considering this level of difference with a two-side significance level of 0.95, power of the study being 0.9, and ratio of cases:controls being 1:2, the minimum sample size was calculated to be 75 in the DWOR group and 150 in the HC group.

Study protocol

All participants underwent the following tests: best-corrected visual acuity (BCVA), IOP by non-contact tonometry (NCT), slit lamp examination, and fundus examination. Spectralis OCT (Heidelberg Engineering, Heidelberg, Germany) was used to acquire OCT images using posterior pole asymmetry scan protocol. Automated retinal segmentation was applied. Scans were checked manually for proper segmentation.

The thickness values of the following retinal layers were noted: RNFL, GCL, IPL, inner nuclear layer (INL), outer plexiform layer (OPL), outer nuclear layer (ONL), retinal pigment epithelial layer (RPE), inner retina (IR), and outer retina (OR).

For each retinal layer, thickness data were noted in the following nine macular sectors: central sub-field (CSF), inner superior (IS), inner inferior (II), inner nasal (IN), inner temporal (IT), outer superior (OS), outer inferior (OI), outer nasal (ON), and outer temporal (OT) within three concentric circles as defined by the Early Treatment Diabetic Retinopathy Study (ETDRS) [10]. Average thickness of the outer ring (Avg_O) was calculated by averaging the thickness values of the four subfields between 3 and 6 mm from the fovea. Similarly, average thickness of the inner ring (Avg_I) was calculated by averaging the thickness values of the four subfields between 1 and 3 mm from the fovea. These measurements were noted for each retinal layer mentioned above.

Retinal networks in HCs and DWORs were calculated as inter-layer covariance matrices using cross-subject correlation of retinal layer thickness.

Retinal structural covariance matrices

Group-based structural covariance networks (SCNs) were estimated for both DWORs and HCs. Adjacency matrices were obtained by calculating the bivariate Pearson's correlation coefficient between the retinal layer thicknesses across the nine quadrants of the seven retinal layers (RNFL, GCL, IPL, INL, OPL, ONL, RPE), with age as a covariate. The resulting absolute of the weighted adjacency matrices of DWORs and HCs were used to estimate the optimal community architecture using Louvain’s modularity.

Community detection and Louvain’s modularity

The optimal community structure is a subdivision of the network into nonoverlapping groups of nodes (retinal subsegments) in a way that maximizes the number of within-group edges and minimizes the number of between-group edges [11]. Modularity is a statistic that quantifies the degree to which the network may be subdivided into such clearly delineated groups.

We used a generalized Louvain-like method [11] originally developed to optimize a single-layer modularity quality function [12] and then extended to optimize the following multilayer modularity quality function [13]:

$𝑄=1/2𝜇\Sigma 𝑖𝑗𝑙𝑟 {(𝐴𝑖𝑗𝑙&minus;𝛾𝑙𝑉𝑖𝑗𝑙)𝛿𝑙𝑟+𝛿𝑖𝑗𝜔𝑗𝑙𝑟}𝛿(𝑔𝑖𝑙,𝑔𝑗𝑟)$

where 𝐴𝑖𝑗𝑙 is the edge between nodes i and j in a time window l of the multilayer network and and 𝑉𝑖𝑗𝑙 is the corresponding element of a null model. Louvain’s modularity (ModL) helps contrast the disruption of the inter-layer community architecture in DWORs compared to HCs.

The input to estimating the community architecture was the absolute group-based SCN, enabling the hierarchical organization of the networks. We chose to use the absolute of the few negative correlations noted in the covariance matrices (which occurred in fewer than 5% of the total number of connections) instead of setting them to zero, because such a correlation does influence the estimation of the graph measures, but the meaning of the effect of its direction in the network remains unclear [14]. We estimated the following outputs: (a) the community assignment (Ci) and (2) modularity Q. Since Ci and Q may vary from run to run, due to heuristics in the algorithm, we ran 50 iterations and used the mode of Ci to determine the community assignment and the mean of Q to determine the modularity. The modularity Q was compared between controls (Qhc) and DOWR (Qdowr) using t-test (p<0.05 was considered significant).

Regression model for age versus HCs and age versus DWORs

Finally, to evaluate how age-related progression of retinal changes varies between DWORs and HCs, we initially estimated the multiple linear regressions using least squares between the thickness of each of the layers and the age using the “regress” function in MATLAB R2020b. Age-related progressive retinal changes between DWORs and HCs were compared using Fisher's r-to-z transform to assess the difference in the regression slopes between DWORs and HCs. False discovery rate correction was used for multiple comparisons.

## Results

Clinico-demographic details

The demographic details of the subjects are shown in Table 1. The mean age ± SD of the participants was 46.06 ± 13.06 years in the HC group and 50.8 ± 8.6 years among the diabetics (p=0.06).

**Table 1:** Demographic profile of the subjects OCT, optical coherence tomography; DWOR, diabetics without retinopathy; IOP, intraocular pressure

		Healthy controls	DWORs
Gender distribution (N)	Males	40	17
	Females	56	25
Age (mean ± SD) (years)	Overall	46.06 ± 13.06	50.8 ± 8.6
	Males	47.82 ± 15	51 ± 9
	Females	44.63 ± 11	50 ± 8.3
Right eyes (N)		78	39
Left eyes (N)		78	39
OCT (mean ± SD) (μm)		533±27	545±35
Corrected IOP (mean ± SD) (mmHg)		15.9±2.8	17±3

Retinal thickness in DWORs versus HCs

The thickness of the following retinal layers was statistically different between the two groups: RNFL_C (HC: 10.16 ± 2.48 µm versus DWOR: 10.85 ± 2.23 µm; p=0.023), INL_Avg_I (HC: 39.9 ± 3.7 µm versus DWOR: 40.9 ± 3.16 µm; p=0.035), OR_C (HC: 89.9 ± 3.8 µm versus DWOR: 88.7 ± 3.6 µm; p=0.017), and OR_Avg_I (HC: 81.4 ± 3.16 µm versus DWOR: 80.5 ± 2.28 µm; p=0.02).

Age-related progressive retinal changes between DWORs and HCs

**Figure 1:**
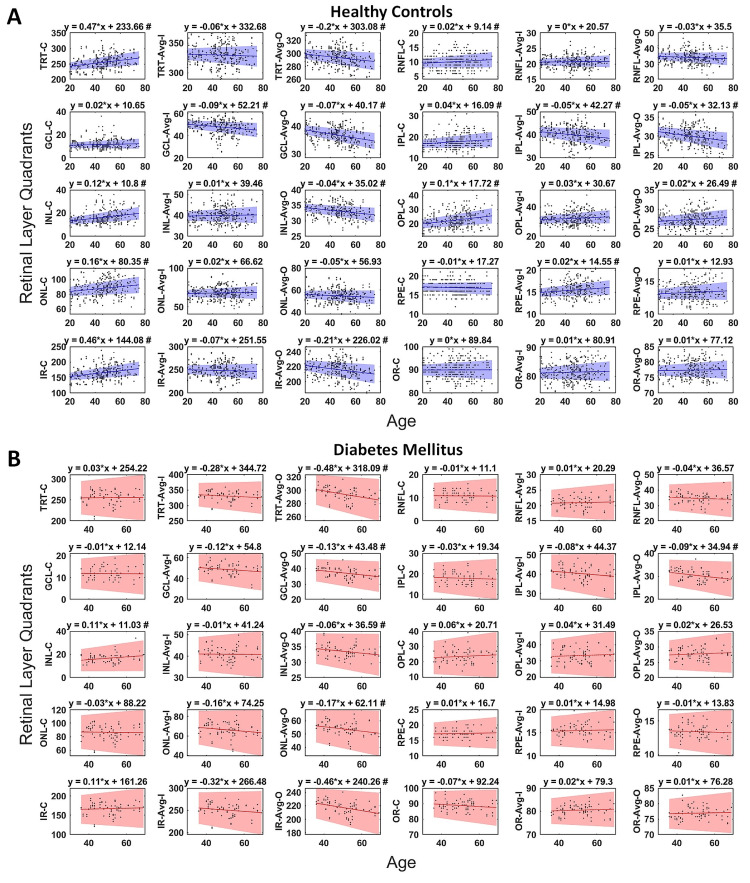
Scatterplots between age (x-axis) and thickness of different retinal layers (y-axis) in the HC and DWOR groups. Regression analysis showing how quadrant-specific thickness changes with advancing age. This was evaluated both in (A) HC and (B) DWOR groups. The x-axis is age, and the y-axis is the quadrant-specific thickness (μm). The regression equations have been provided at the top of the graphs. #next to the equation implies a significant correlation between quadrant-specific thickness and age. HC, healthy control; DWOR, diabetics without retinopathy

The CSF showed an age-related thickening in RNFL (r=0.117, p=0.04), GCL (r=0.078, p=0.17), IPL (r=0.137, p=0.01), INL (r=0.29, p≤0.001), OPL (r=0.256, p<0.001), and ONL (r=0.197, p=0.001) layers in HCs. In the DWOR group, a similar thickening with age was seen only in the INL layer (r=0.244, p=0.03). The RFNL_C quadrant showed a thinning with age in the DWOR group as against thickening noted in the HC group, which was statistically significant (r-value of DWOR [rDWOR] =-0.023, r-value of HC [rHC]=0.12, z=2.6, p=0.009). The overall thickness of the retina in the CSF (TRT_C) showed greater thickening with age in the HC group compared to the DWOR group (rDM =0.016, rHC=0.28, z=2.07, p=0.03). The IR layers (GCL and IPL) showed an age-related thinning in all the inner and outer ring sectors in the HC group (r’s > -0.149, p’s < 0.016). Also, an age-related thickening of the RPE was observed in all the sectors of the inner ring among HCs. (r's > 0.08, p's < 0.005) However, in the DWOR group, age-related thinning of GCL/IPL was observed only in the inferior, temporal, and nasal sectors of the outer ring, and no change with age was seen in the RPE layer. The TRT_ON quadrant showed greater thinning with age in the DWOR group compared to HCs, which was statistically significant (rDM = -0.49, rHC = -0.22, z=2.34, p=0.019). Figure 1 depicts the scatterplots between age (x-axis) and thickness of different retinal layers (y-axis) in the HC and DWOR groups.

Disrupted modular architecture of retinal layers in DWORs compared to HCs

**Figure 2:**
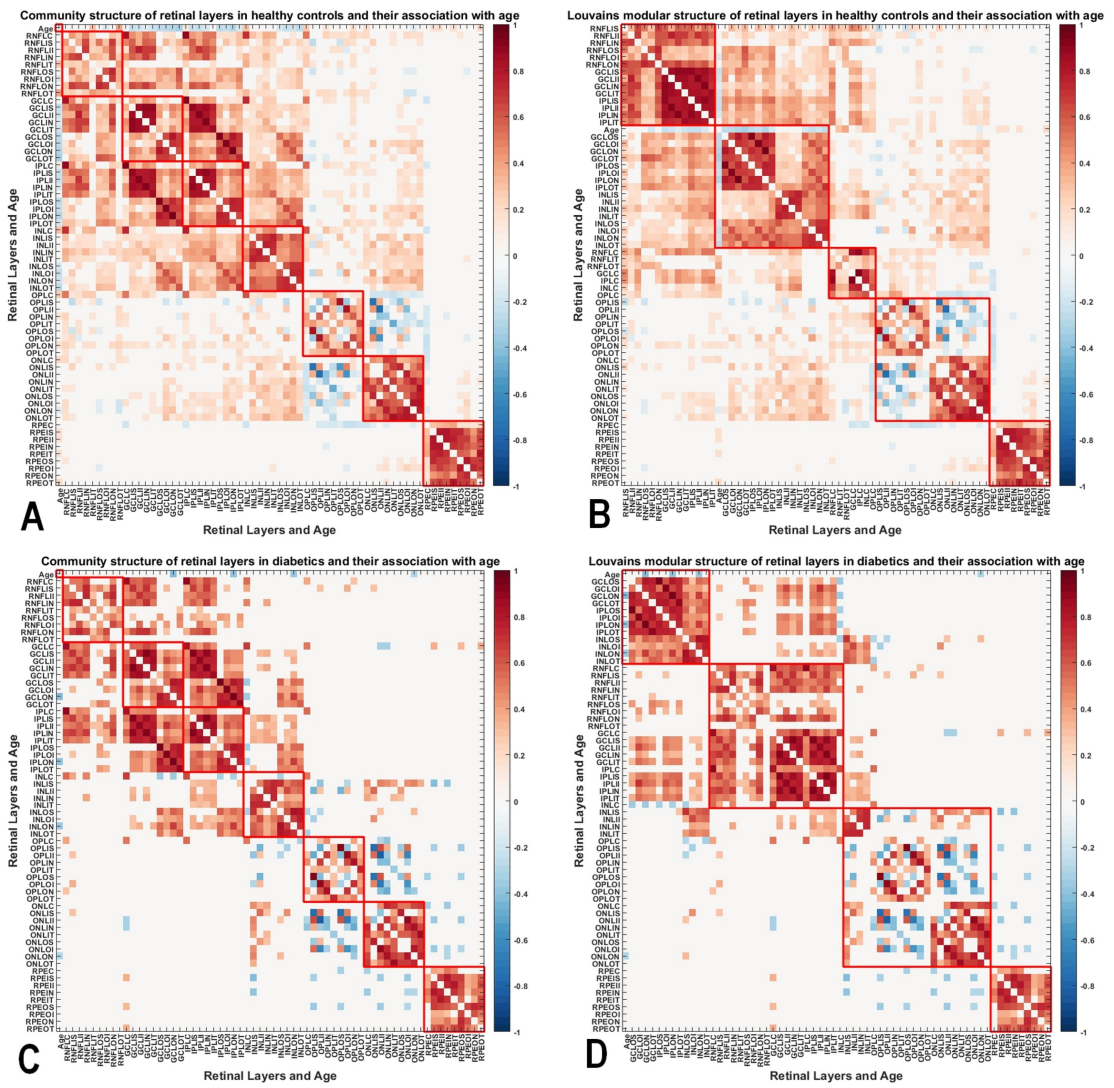
Modular architecture of retinal layers in DWORs compared to HCs (A) This covariance graph shows the original structural relationship between various retinal layer thicknesses. These original communities are assigned based on the quadrant per given retinal later. (B) Louvain's community detection algorithm was implemented to detect optimal communities from this large network structure, such that each community had retinal layers grouped based on the highest interregional covariance with minimal between-community interactions. This implies that these regions are most likely to have retinal thickness changes in congruence with each other. In HCs, at gamma set to 1, we noted five distinct communities. (C) In the DWOR group, we note a disruption in the community architecture. Unlike in HCs, we noted minimal inter-community interactions in the DWOR group. (D) After testing Louvain's modularity, due to a more homogenous pathological change in retinal thickness, we noted only four communities with a marked decrease in intercommunity interactions. This implies that in pre-clinical diabetic retinopathy, some retinal layers and quadrants are affected early and tend to have similar pathological organization early in the disease process. HC, healthy control; DWOR, diabetics without retinopathy

There was an abnormal increase in modularity among DWORs compared to HCs (Qhc=0.47, Qdowr=0.51, p=1.6x10^-8^). In the DWOR group, we noted a disruption in the community architecture. Unlike in HCs, we noted minimal inter-community interactions in the DWOR group. After testing Louvain's modularity, due to a more homogenous pathological change in retinal thickness, we note only four communities with a marked decrease in inter-community interactions in the DWOR group (Figure 2).

## Discussion

Our study demonstrates significant differences in the thickness of retinal layers in the DWOR group compared to the HC group in the form of greater thickness of IR (RNFL_C and INL_Avg_I) and thinning of OR (OR_C and OR_Avg_I) in the DWOR group compared to the HC group. The INL is mainly formed by the nuclei of bipolar cells and Muller cells, and Muller cell activation and hypertrophy have been noted in early DR [15,16]. This could explain the thickening of INL in DWORs. DR predominantly affects inner retinal vasculature causing pericyte loss. This combined with increased capillary leakage results in diabetic macular edema. However, the influence of this on retinal thickness in patients with no DR has not been studied. From our findings, we can postulate that subclinical thickening of IR happens in early DR process. Our findings are consistent with the findings of Vujosevic and Midena [4], wherein a similar thickening of INL was found in the diabetic group. Van Dijk et al. [5,6] showed that RNFL, GCL, and IPL were thinner in patients with minimal DR compared to controls. This raises the possibility that in pre-clinical stage of DR, the inner retinal thickness increases owing to sub-clinical edema and Muller cell hypertrophy, and once mild NPDR sets in, there is thinning owing to cellular degeneration.

We also found a decrease in outer retinal thickness in DWOR compared to HC. Verma et al. also showed that photoreceptor thickness at the fovea was reduced in diabetics compared to controls due to neurodegeneration [17].

Structural covariance analysis has long been used in studying neurological diseases [18,19]. It is a multivariate analysis technique that conceptualizes how the morphological properties of different brain regions relate to each other at the group level. Anatomically and developmentally, the retina is an extension of the brain. Many neurodegenerative diseases that affect the brain have retinal manifestations. Hence, an attempt has been made to apply the same concepts of structural covariance analysis to retinal networks. We found that there was an abnormal increase in modularity in the DWOR group compared to the HC group. This indicates a disruption of inter-retinal layer interaction in the DWOR group compared to the HC group.

This study has limitations. The DWOR group of patients was selected based on clinical examination only and not on fluorescein angiography or vitreous fluorometry findings, which would have helped define the study population better. The systemic control of blood sugars was not taken into consideration during case selection. The ideal way to assess age-related changes in the thickness of retinal layers would be a longitudinal study with serial measurements in the same patients. However, we have drawn conclusions based on cross-sectional data rather than longitudinal data. Subjects of Indian ethnicity alone were included.

The results of this paper add to the growing body of evidence on the pathogenic mechanisms in pre-clinical DR. In vitro studies designed to understand the changes at a cellular level are necessary to substantiate the results of this study. Our results provide the primer to developing a concept of network-level understanding of retinal layer functions in health and disease.

## Conclusions

RNFL and INL are thicker in DWOR compared to HC. Outer retinal layers are thinner in DWORs compared to HCs. On modularity analysis, we noted a disruption in the community architecture in the DWOR group compared to the HC group. Currently, there is no definite way of reversing the changes of DR once it is clinically manifest. Hence, diagnosis of DR in the pre-clinical stage may help find solutions to reversing DR changes in the retina. Future research should be directed at developing therapies that address both neural and vascular damage. Advances in neuroprotection and neuroregeneration, alongside better vascular treatments, might create a comprehensive treatment strategy that addresses the full spectrum of damage caused by diabetes.
